# Measuring the process and rate of exogenous DNA degradation during digestion in mice

**DOI:** 10.1038/s41598-022-10340-7

**Published:** 2022-04-19

**Authors:** Ruiqi Xing, Hui Liu, Xia Qi, Lingzi Pan

**Affiliations:** 1 Second Affiliated Hospital of Dalian Medical University, Dalian, China; 2 College of Medical Laboratory, Dalian Medical University, Dalian, 116044 China; 3 Dalian Blood Center, Dalian, China

**Keywords:** Molecular biology, Physiology, Zoology

## Abstract

This study aimed to perform qualitative and quantitative examination of DNA degradation during the digestion process in the mouse gut through PCR, qPCR and short tandem repeat (STR) analysis. Human blood leukocytes were gavaged into the digestive tract in mice. GAPDH, TH01, TPOX and D7S820 genes in the contents of the stomach and small intestine were analyzed with PCR and qPCR at various times pre- and post-gavage. Through STR analysis, 21 human genomic DNA loci were analyzed. The half-life of DNA degradation, and the relationship between the average peak area and digestion time were determined. The PCR results showed bands of amplified genes at pre-gavage (0 min) and post-gavage (40, 80 and 120 min) from the mouse stomach contents, whereas no DNA bands from small intestinal chyme were observed after gavage. The qPCR results revealed a significant decrease in DNA concentrations during 40–120 min in the mouse stomach after gavage. At 120 min, 85.62 ± 8.10% of the DNA was degraded, and the half-life of exogenous DNA degradation in the mouse stomach was 70.50 ± 5.46 min. At various digestion times, almost no target genes were detected in the mouse small intestinal chyme. STR analysis showed a decrease in allele numbers with bowel advancement in the small intestine in mice. The degradation of exogenous DNA was higher in the mouse stomach during the first 2 h, and almost complete degradation was observed within 40 min after entering the small intestine in mice.

## Introduction

Deoxyribonucleic acid digestion and absorption in the gastrointestinal tract (GIT) has been a topic of great interest. Studying the fate of DNA in the digestive tract can be used to assess the risk associated with food and medicine entering the body. Vieira^1^ has used PCR to detect human blood DNA in the digestive tract of *Aedes aegypti* mosquitoes, thus facilitating epidemiological investigation and forensic identification.

Dietary DNA in the digestive tract has been thought to be completely hydrolyzed by digestive enzymes and acids in the mouth and GIT. However, this assumption was made without analysis of the sources or the remaining DNA fragments; instead, it was based on measurement of the biochemical degradation of DNA into single base pairs^2,3^. Subsequently, DNA was observed to degrade more rapidly in the upper part of the gastrointestinal tract than in the lower part, and plasmid DNA extracted from the intestinal contents of rats 5 h after gavage has been found to be biologically active^4^. However, Loretz has reported that pig gastric juice and intestinal fluid almost completely degrade plasmid DNA within 1 hour^5^. Rabbit DNA has been found in the blood of two volunteers who ate 400–600 g of cooked rabbit meat^6^. DNA fragments have been observed in chicken muscle, liver, spleen and kidney^7,8^. Similarly, soybean DNA fragments have been detected in the GIT, tissues and other organs of pigs, sheep and cattle^9–11^. However, Walsh^12^ has detected cry1Ab genes and proteins in the digestive fluids but not the tissues (i.e., kidney, liver, muscle, heart or blood) of pigs fed Bt MON810 corn. Sattarzadeh et al.^13^ reported the presence of the Nptll gene and Nos promoter sequences in the stomach, but not other tissues, in F-DT and M-LT rats. Trojan reported no detection of purple wheat genes in the blood of chickens, rats and carp fed purple wheat^14^. Nawaz et al.^15^ found that food DNA can be digested into DNA fragments up to several hundreds of bp, as detected in the GIT. Various researchers have reported similar observations of dietary DNA not only in the GIT in humans or animals, but also in the blood, other tissues or even various organs. The fate of dietary nucleic acids in animals has long been an exciting and controversial research topic.

Digestion of nucleic acids is generally believed to occur in the intestines^16^, the main site of digestion and absorption of nutrients and nucleic acids, the latter of which are hydrolyzed by nuclease, phosphodiesterase, alkaline phosphatase and nucleosidase into oligonucleotide-single nucleotide-non-nucleoside bases in the intestine^3,15^. Gastric juice consists of pepsin and gastric acid. Pepsin’s main function is protein digestion, but in recent years, Liu^17^ found that pepsin in gastric juice can digest not only protein but also nucleic acids. The digestion of nucleic acids starts in the stomach, and various animal pepsins have different abilities to digest nucleic acids^18^. Most dietary DNA is in the form of histones, which form nucleosomes. The complex components of the diet may affect the digestion of DNA by pepsin. Zhang^19^ has demonstrated that common food components, including proteins, carbohydrates, metal cations and polycationic compounds, affect the digestion of DNA through in vitro simulation studies. Therefore, the digestion of DNA from different sources in animals requires further analysis.

The fate of DNA in the GIT in animals could be used as a model to assess the risk and efficacy of drugs and foods entering the body. The fate of digested DNA and the degradation rates of DNA from various sources in the GIT in different animals have not been studied in detail. Most researchers have studied the degradation of DNA in the GIT of various animals either in extracted animal gastric and intestinal fluids or through in vitro simulation models. In in vitro studies, many factors must be controlled, such as temperature, pH, ionic strength, enzyme concentration, GIT flora and epithelium. Mice have been used as a model to explore the dietary DNA degradation and digestion process in the mouse GIT at various time intervals. The DNA degradation rate has been quantitatively analyzed.

At present, study of dietary DNA traceability has been mainly based on the polymerase chain reaction (PCR) technique, and the minimum amplifiable fragment length is 70–100 bp^20,21^. Short tandem repeat (STR) sequences are a series of small DNA fragments of 2–7 bp repeats present in approximately 10–60 copies in the human genome. STR loci are highly polymorphic and widely distributed. The range of allele fragments is small, and simultaneous detection of multiple loci can be performed in STR analysis, which can also decrease the loss of alleles due to the dominant amplification of small fragments. The target fragment after PCR amplification is small, and the smallest detectable fragment is 77 bp; therefore, STR analysis is suitable for the detection of degraded DNA^22^.

In this study, STR analysis, which is commonly used in human forensic identification, was used as a marker to observe the DNA degradation in the gastrointestinal tract in mice. The STR genes used in human forensic identification have good stability and are not easily damaged. These genes also have the advantage of forming small gene fragments and having advanced detection technology available, thus, aiding in the detection of damaged DNA present in small fragments in the stomach and small intestine in mice, and increasing the reliability of the results. STR loci are usually not present in animals and food, and are very common in forensic identification. Therefore, human blood genomic DNA was chosen as an exogenous gene to infuse into the digestive tract in mice. PCR, qPCR and STR analyses were used to qualitatively and quantitatively evaluate the exogenous DNA degradation rate and half-life in the gastrointestinal tract in mice at various time intervals.

## Materials and methods

We confirm that all methods were performed in accordance with the relevant guidelines and regulations, and all experimental protocols were approved by the University Ethics Committee of Dalian Medical University, under ethics number AEE18036.

**Figure 1:**
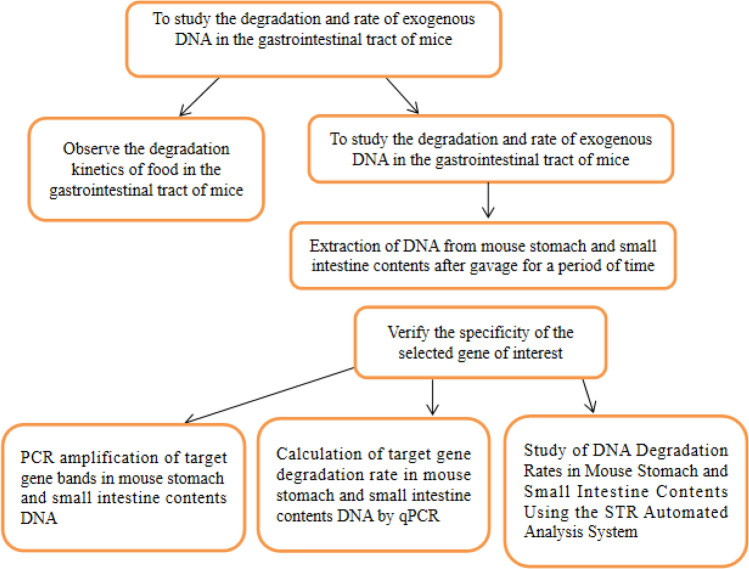
Experiment mind map.

The mind map of the entire study is shown in Fig. 1.

### Experimental animals

Kunming mice were obtained from the Experimental Animal Center of Dalian Medical University. The mice weighed approximately 17–30 g, and both sexes were included.

### Observation and calculation of the small bowel advancement rate

#### Preparation of nutritive semi-solid paste

Previously described methods were followed^23^ with slight modifications. A total of 5 g sodium carboxymethylcellulose was dissolved in 125 mL distilled water. Then the following were added: 8 g milk powder, 4 g sugar, 4 g starch and 2 g activated carbon. The mixture was stirred gently, and 150 ml containing approximately 150 g of a black semisolid paste was prepared. The product was stored at 4 °C and was brought to room temperature before gavage.

#### Gavage in mice

After fasting for 24 h, the mice were gavaged with a nutritional semi-solid paste (0.8 ml) and water.

#### Mouse sacrifice and dissection

Three mice per group were sacrificed by cervical dislocation at the following time intervals: 10, 20, 30, 40, 50, 60, 70, 80, 90, 120, 150, 180, 360, 420, 510, 540 or 570 min. Through incision of the abdominal cavity, the stomach and intestine were separated. The small intestine was measured from the pylorus to the ileo-cecal region (d1). The distance from the pylorus to the mid-point of the black semi-solid paste was measured (d2). The small bowel advancement rate was calculated as:

$$\frac{{{\text{d2}}}}{{{\text{d1}}}} \times {{100\% }}$$

### Analysis of the process and rate of exogenous DNA degradation in the mouse gastrointestinal tract by PCR, qPCR, and STR assays

#### Preparation of the buffy coat layer

A total of 35 ml of human EDAT whole blood was obtained from a healthy volunteer donor at Dalian Medical University, and informed consent was obtained. Blood samples were centrifuged at 5000 g for 10 min. Plasma was discarded, and the buffy coat layer was carefully collected and stored at 4 °C.

#### Gavage and DNA extraction

Nutritive semi-solid paste was prepared as described above, except with the addition of 2 g of activated carbon powder. A total of 0.4 ml semi-solid nutrient paste was mixed with the buffy coat (0.4 ml). The nutrient semi-solid paste was administered to mice by gavage. Mice were sacrificed by cervical dislocation at time intervals of 0, 40, 80, or 120 min. Through incision of the abdomen, an equal amount of stomach and small intestine content was collected in a 1.5 ml EP tube. DNA was extracted according to the instructions of the E.Z.N.A. blood DNA kit (Beijing Solarbio).

#### Screening of exogenous target genes

In this experiment, human blood genomic DNA was used for exogenous target gene. The non-homologous human housekeeping gene GAPDH, TH01^24^, TPOX and D7S820 (Table 1) were obtained from Dalian Ruizhen Biotechnology and Suzhou Gema Gene as the target genes respectively. PCR primers for GAPDH, TH01, TPOX and D7S820 were used to amplify human blood DNA and mouse liver DNA. The amplification reaction was performed in 25 μl volumes containing 2 μl template DNA, 0.5 μl each of forward and reverse primers, 12.5 μl of 2 × Power Taq PCR Master Mix and 9.5 μl of deionized water. Thermal cycling was conducted under the following conditions: initial denaturation step (94 °C; 60 s), denaturation (94 °C; 30 s), annealing (51 °C; 30 s) and extension (72 °C; 30 s), for a total of 35 cycles. The products were electrophoresed on a 2% agarose gel to observe the amplified target genes. The results of the agarose gel electrophoresis were analyzed with a UVPC-80 gel imaging system (UCP Inc., San Jose, CA, USA).

**Table 1:** Four target gene primer sequences.

Primer name	Primer sequence (5′ to 3′)	Target gene fragment length (bp)
GAPDH upstream	AGTGGAAGACAGAATGGAAGAAATG	106
GAPDH downstream	TGGGGACAGGACCATATTGAG	
TH01 upstream	ATTCAAAGGGTATCTGGGCTCTGG	234
TH01 downstream	GTGGGCTGAAAAGCTCCCGATTAT	
TPOX upstream	TGCGTAATCCTCCACTAACTGA	79
TPOX downstream	TCCAACGGGAATGGCTCT	
D7S820 upstream	CACCTGTTACCTCCAGTTTCC	77
D7S820 downstream	TTTGCTGCTTTAGTCTTCCTTC	

#### Qualitative analysis of target genes by PCR

The DNA extracted in step 2.5.2 was amplified by PCR, with the detection steps as described above.

#### qPCR determination of the kinetics of DNA degradation in the digestive tract in mice

The extracted DNA samples were amplified. The amplification reactions were performed in 20 μl volumes containing 2 μl of template DNA, 0.4 μl each of forward and reverse primers, 10 μl of 2 × ChamQ Universal SYBR qPCR Master Mix (Vazyme Biotech Co., Ltd.) and 7.2 μl of ddH2O. The reaction conditions included pre-denaturation (95 °C, 30 s), denaturation (95 °C, 5 s) and annealing (60 °C, 45 s); after 40 cycles of amplification, the results of the reaction were detected with a qPCR instrument MA-6000 (Molarray, Suzhou, China) and analyzed.

#### STR determination of the kinetics of DNA degradation in the digestive tract in mice

The extracted DNA was analyzed through STR typing detection with an AGCU Expressmarker 22 Fluorescence Detection Kit (Wuxi Zhongde Meilian Biotechnology Co., Ltd.). A total of 38 alleles at 21 loci were amplified (Table 2). According to the instructions, each PCR amplification was performed in a 10 μl reaction. The amplification conditions were pre-denaturation (95 °C, 2 min), denaturation (94 °C, 30 s), annealing (60 °C, 1 min) and extension (70 °C, 1 min), for 10 cycles of amplification; then denaturation (90 °C, 30 s), annealing (58 °C, 1 min) and extension (72 °C, 1 min), for 20 cycles of amplification and extension for 10 min at 72 °C. After amplification, 1 μl of PCR product was loaded onto an ABI 3130 Avant Genetic Analyzer (Forster, California Applied Biosystems) for capillary electrophoresis, and GeneMapper®ID software v3.2 (Applied Biosystems, Foster City, California) was used to analyze the data. Allele peaks were labeled when the peak area was ≥ 50 relative fluorescence units (RFU). The relative concentration of DNA degradation was calculated according to the STR spectrum and peak area generated by the software.

**Table 2:** Post gavage (0 min) STR analysis: loci, alleles and peak areas.

Locus	Allele		Peak area		Locus	Allele		Peak area	
D19S433	13	14	51595	44400	Penta D*	9	9	67023	–
D5S818	11	13	45242	40189	D2S441	10	11	71981	59984
D21S11	30	32.2	39591	43919	vWA	15	16	82882	78403
D18S51	13	19	37318	29596	D8S1179*	15	15	110223	–
D6S1043	18	19	34582	31183	TPOX	8	12	116575	104151
AMEL*	X	X	65353	–	Penta E	11	17	106722	84672
D3S1358	16	18	28865	27571	TH01	6	7	101214	94798
D13S317	8	12	46242	36760	D12S391	19	24	52279	46793
D7S820*	11	11	88244	–	D2S1338	23	25	113265	48908
D16S539	10	13	48549	51712	FGA	21	22	64963	57056
CSF1PO	10	12	45441	41806	–	–	–	–	–

Average peak area = (51595 + 44400 + 45242 + 40189 + 39591 + ⋯ + 64963 + 57056)/38 = 61580.26.

*Represents homozygote.

### Data analysis

#### Relative quantification of DNA through qPCR

According to the qPCR amplification principle, PCR products increase exponentially as they are amplified. If the amplification efficiency was “E”, and the initial quantity of template was “N0,” then the amount of PCR product after “m” cycles was “Nm,” according to the formula ${\text{Nm = N}}_{{0}} {\text{(1 + E)}}^{{\text{m}}}$. The initial quantity of template pre-gavage was recorded as “N0” after “Ct0” cycles, and reached the baseline “Nq,” while the initial quantity of template at different times after gavage was recorded as “Nn” at “Ctn” cycles, and reached the baseline “Nq.” Therefore, $N0\left( {1 + E} \right)^{Ct0} = Nn\left( {1 + E} \right)^{Ctn}$. We assumed that the amplification efficiency was 1. Then, according to the formula $\frac{Nn}{{N0}} = 2^{{\left( {Ct0 - Ctn} \right)}}$, the relative quantity of DNA in the stomach in mice at different digestion times was calculated.

#### STR analysis

**Figure 2:**
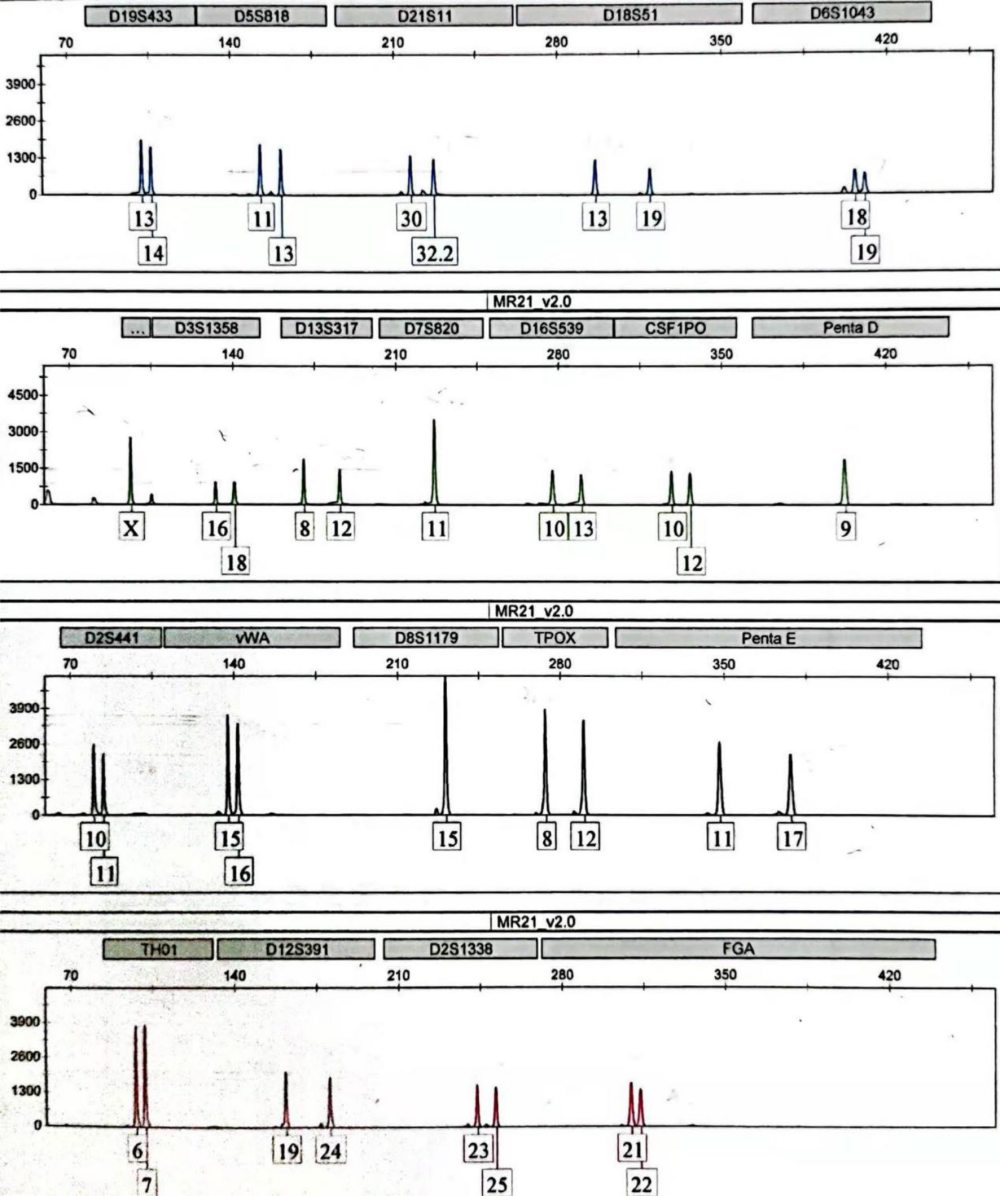
STR map of a sample.

The relative concentrations of degraded DNA, expressed as the mean peak area, was calculated by dividing the sum of the interpreted peak areas by the total peak number of the full STR profile (Table 2). STR patterns (Fig. 2) amplified from gastric content DNA, 0 min after gastric gavage are shown as an example to illustrate the calculation method of the average peak area.

Linear regression analysis was performed. The slope (K) was calculated between the natural logarithm (ln) of the average peak area and the digestion time. The half-life of DNA degradation in the mouse stomach was determined with the following formula^25^:

$$T\raise.5ex\hbox{$\scriptstyle 1$}\kern-.1em/ \kern-.15em\lower.25ex\hbox{$\scriptstyle 2$} = \ln 2/\left| k \right|$$

## Results

### Advancement rate of nutritional semi-solid paste in the GIT in mice

**Figure 3:**
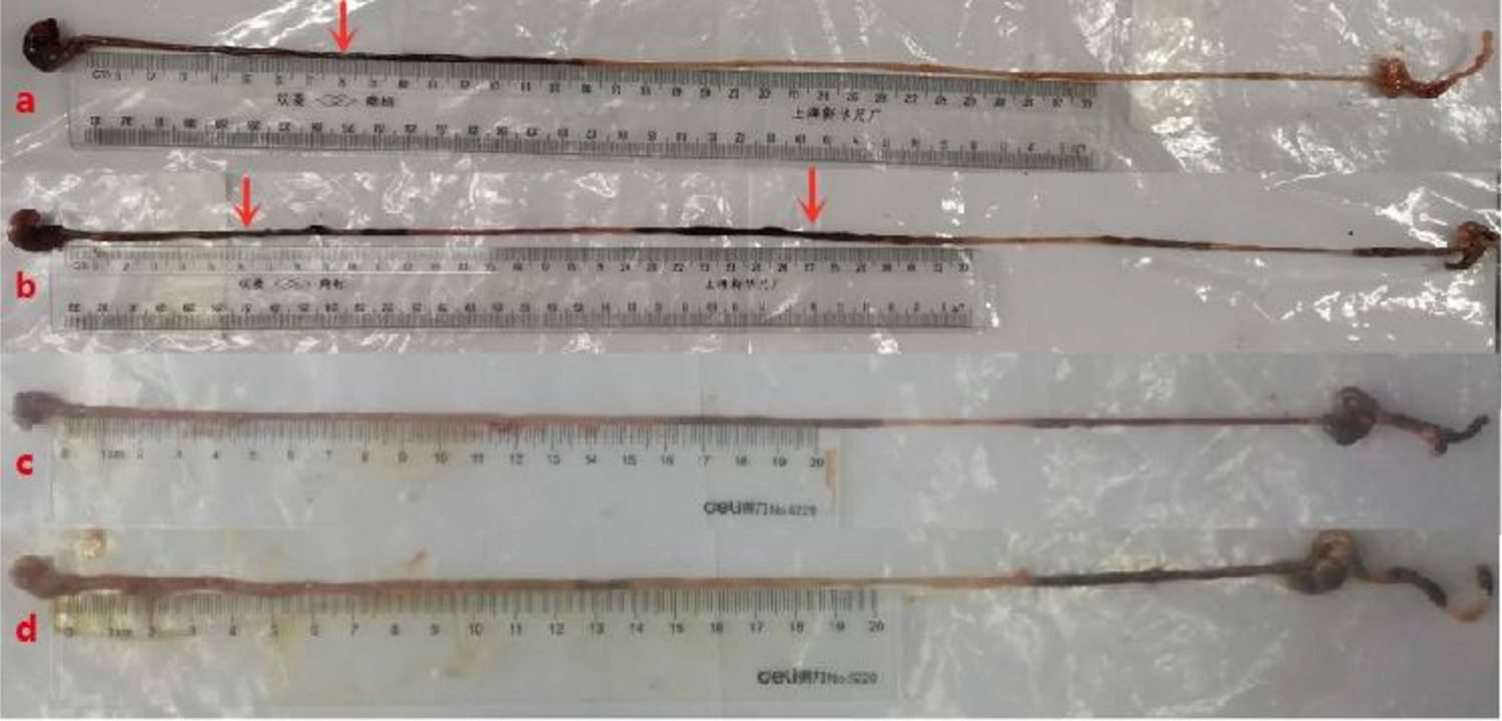
The emptying of nutritious semi-solid paste in the gastrointestinal tract in mice at different digestion times. From left to right, the image shows the mouse stomach, small intestine and cecum; black represents the location of the nutritional semi-solid paste. (**a**) GIT of mice at 10 min after gavage. (**b**): GIT of mice at 20 min after gavage, (**c**) GIT of mice at 360 min after gavage. (**d**): GIT of mice at 420 min after gavage.

**Figure 4:**
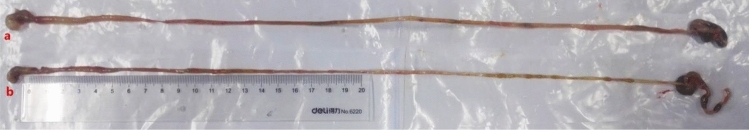
At 540 min after gavage, the nutritive semi-solid paste was completely emptied from the stomach and small intestine in mice. (**a**) and (**b**) are GIT images from mice at 540 min and 570 min after gavage. Whole nutritional semi-solid paste in the stomach and small intestine entered the cecum.

Figure 3 and Fig. 4 show the positions of the nutritive semi-solid paste in the GIT in mice at various times after gavage (black indicates the position of the nutrient semi-solid paste). The semi-solid paste in the stomach and intestine in mice was observed, and the intestinal advancement rate was measured (Table 3). At 10 min after gavage, the intestinal advancement rate was 20.27 ± 2.60%. The mouse stomach was completely emptied in approximately 360 min. The stomach and small intestine were completely emptied in approximately 540 min, and all the semi-solid paste entered the cecum.

**Table 3:** Digestion time, length of small intestine and small bowel advancement rate of nutrient semi-solid paste in the GIT in mice.

Digestion time (min)	Length of small intestine (cm)	Small bowel advancement rate (%)				Surplus in stomach
		First stage	Second stage	Third stage	Fourth stage	
10	34.75 ± 1.48	20.27 ± 2.60	–	–	–	+
20	32.27 ± 7.18	9.70 ± 6.43	31.86 ± 9.76	–	–	+
30	35.83 ± 4.80	11.77 ± 8.11	44.05 ± 16.66	–	–	+
40	38.90 ± 1.98	15.93 ± 5.55	47.77 ± 12.11	–	–	+
50	37.10 ± 1.41	18.74 ± 6.91	56.01 ± 16.93	–	–	+
60	36.45 ± 2.76	48.47 ± 3.18	89.59 ± 7.56	–	–	+
70	41.60 ± 5.60	38.76 ± 5.11	59.20 ± 8.80	88.20 ± 8.45	–	+
80	40.85 ± 4.88	47.53 ± 3.50	72.33 ± 7.62	88.70 ± 5.40	–	+
90	45.10 ± 2.40	31.68 ± 5.22	63.44 ± 5.11	89.51 ± 5.55	–	+
120	45.70 ± 1.70	–	–	79.09 ± 0.78	100	+
150	52.95 ± 1.91	30.18 ± 12.93	72.37 ± 11.21	97.10 ± 4.11	100	+
180	57.25 ± 4.45	44.59 ± 6.72	65.58 ± 7.00	85.83 ± 8.58	98.99 ± 1.44	+
360	36.25 ± 2.75	–	56.12 ± 8.66	87.61 ± 12.39	100	−
420	36.03 ± 2.66	–	–	81.44 ± 10.42	100	−
510	45.45 ± 3.18	–	–	67.18 ± 2.69	100	−
540	43.55 ± 5.30	–	–	–	100	−
570	38.40 ± 0.57	–	–	–	100	−

“+” Surplus in stomach. “–” None remaining in stomach.

### PCR results of exogenous DNA target genes

**Figure 5:**
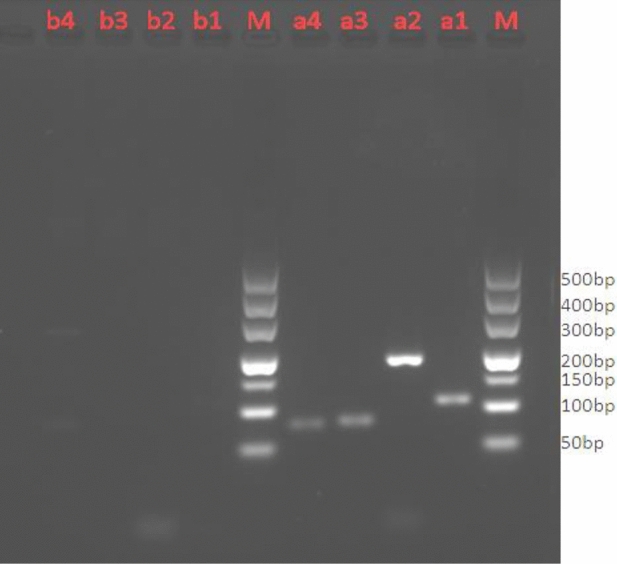
Verification of the specificity of the target gene. Lane M: 500 bp DNA marker, lanes a1–a4: human blood DNA (template DNA used to amplify the target genes GAPDH (106 bp), TH01 (234 bp), TPOX (79 bp) and D7S820 (77 bp), with clear amplified bands. Lanes b1–b4: mouse liver DNA (template used to amplify the target genes GAPDH, TH01, TPOX and D7S820), with no amplified bands.

The selected exogenous target genes GAPDH, TH01, TPOX and D7S820 were analyzed for homologous sequences. DNA bands were detected for human target genes, whereas no bands were detected for mouse genes (Fig. 5).

### PCR results of DNA degradation in the mouse GIT

**Figure 6:**
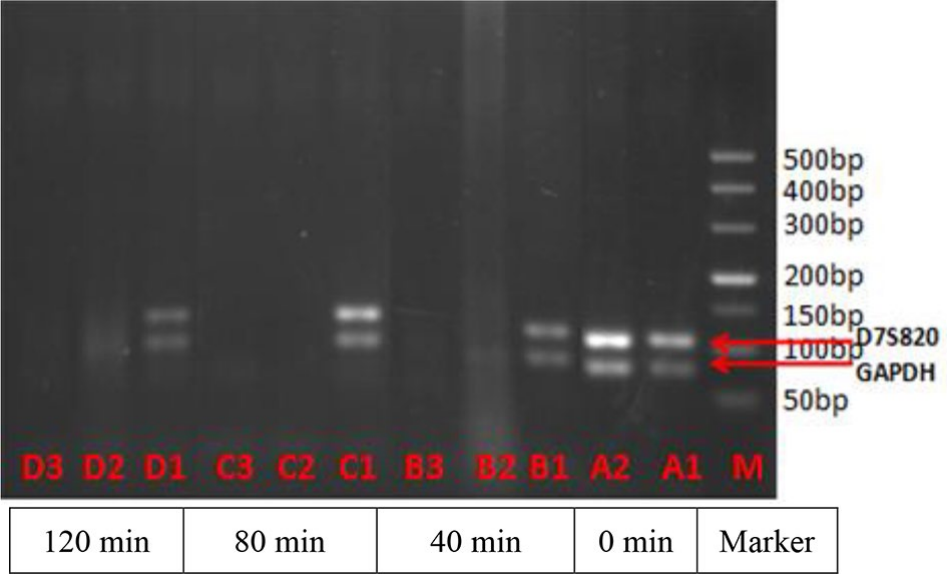
Electrophoresis results of GAPDH and TH01 in DNA in the mouse stomach and small intestine contents. Lane M: 500 bp DNA marker. Lanes A1 and A2: pre-gavage (0 min). Lanes B1–B3, C1–C3 and D1–D3: post-gavage at 40, 80 and 120 min, respectively. Lanes B1, C1 and D1: DNA of stomach contents, with GAPDH (106 bp) and D7S820 (77 bp) bands. Lanes B2, B3, C2, C3, D2 and D3: DNA of small intestine contents, with no amplified DNA bands.

**Figure 7:**
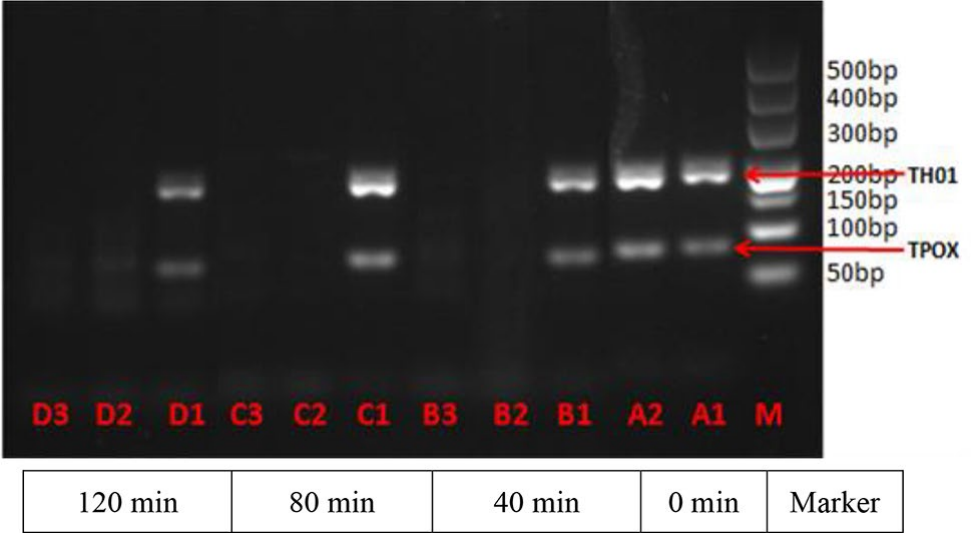
Electrophoresis results of GAPDH and TH01 in DNA in the mouse stomach and small intestine contents. Amplification of the TH01 (234 bp) and TPOX (79 bp) genes. Lane M: DNA marker. Lanes A1 and A2: pre-gavage (0 min). Lanes B1–B3, C1–C3 and D1–D3: post-gavage times of 40, 80 and 120 min, respectively. Lanes B1, C1 and D1: stomach content DNA bands. Lanes B2, B3, C2, C3, D2 and D3: DNA of small intestine chyme, with no DNA bands.

The results of PCR in Fig. 6 revealed the target DNA bands for GAPDH, D7S820 (i.e., lanes A1, A2, B1, C1 and D1) in the stomach contents at various times (0–120 min) pre and post gavage. No DNA bands were observed in the small intestine contents (i.e., lanes B2, B3, C2, C3, D2 and D3) at 40–120 min after gavage, as shown in Fig. 6. The TH01 and TPOX gene amplification results (Fig. 7) showed the presence of the target DNA bands in lanes A1 and A2 (0 min; pre-gavage); lanes B1, C1 and D1 represent the stomach contents and showed clear bands at 40, 80 and 120 min post-gavage, respectively. No DNA bands (TH01 and TPOX) were observed in the chyme obtained after 40–120 min from the small intestine (i.e., lanes B2, B3, C2, C3, D2 and D3) at various times after gavage, as shown in Fig. 7.

### qPCR results of DNA degradation in the mouse GIT

**Figure 8:**
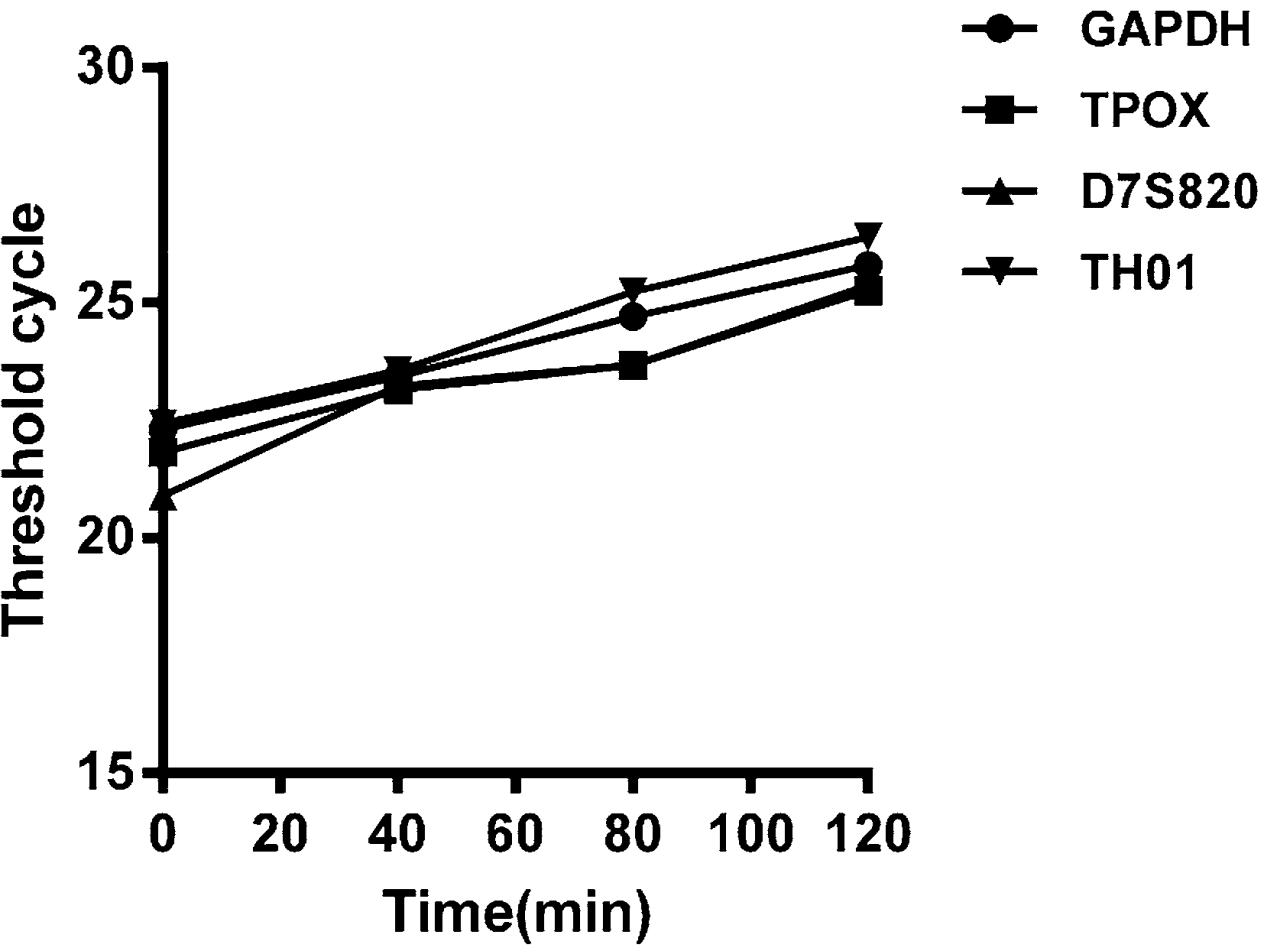
Ct values of four target genes at various times.

The GIT contents were also analyzed through qPCR for the target genes (i.e., GAPDH, TH01, TPOX and D7S820) pre-gavage (0 min) and at different times post-gavage (i.e., 40, 80 and 120 min). The four target genes were amplified, and each group was analyzed three times. Pre and post-gavage times and the Ct values of four target genes are plotted in Fig. 8.

**Figure 9:**
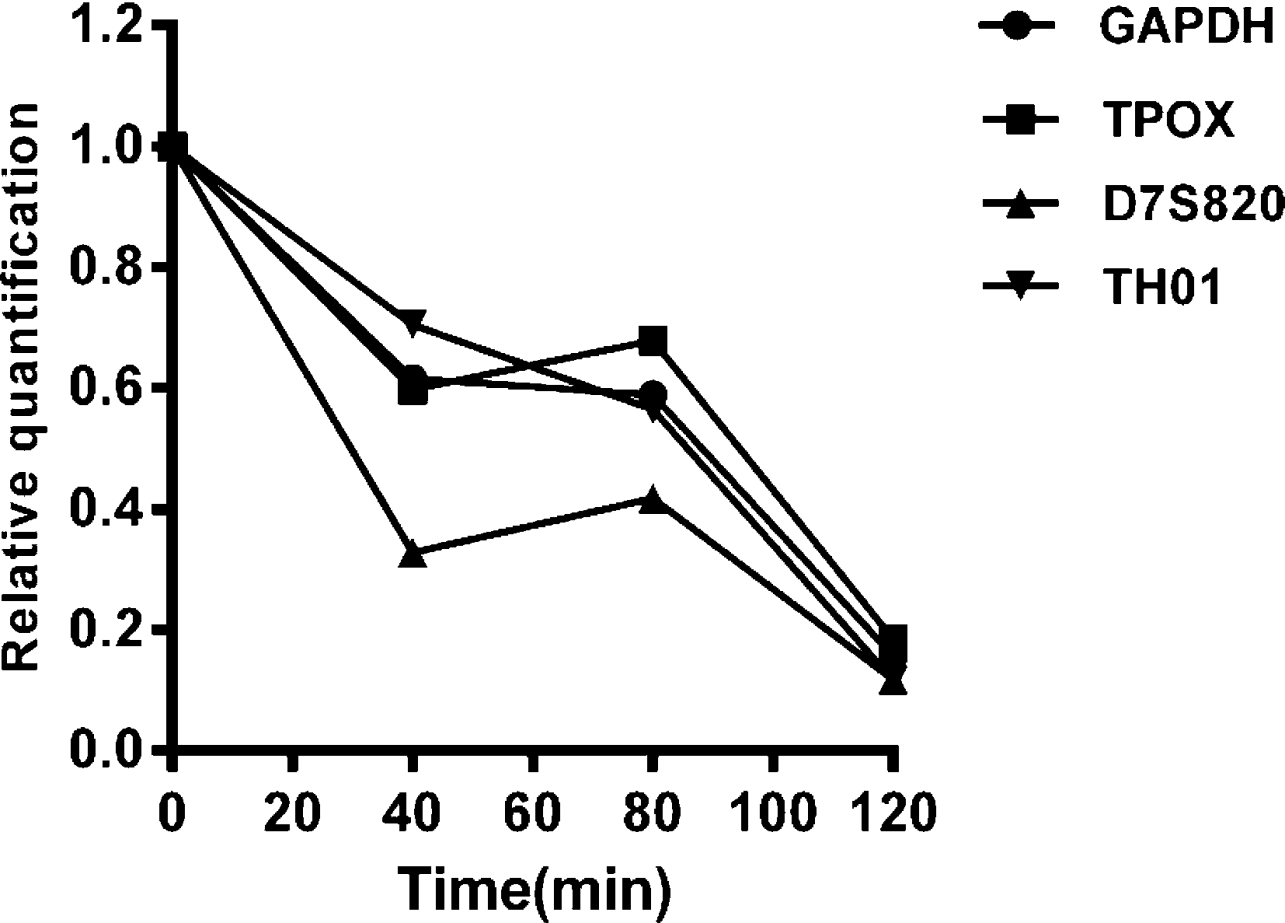
Relative Ct values of the four targeted genes at different digestion times.

**Figure 10:**
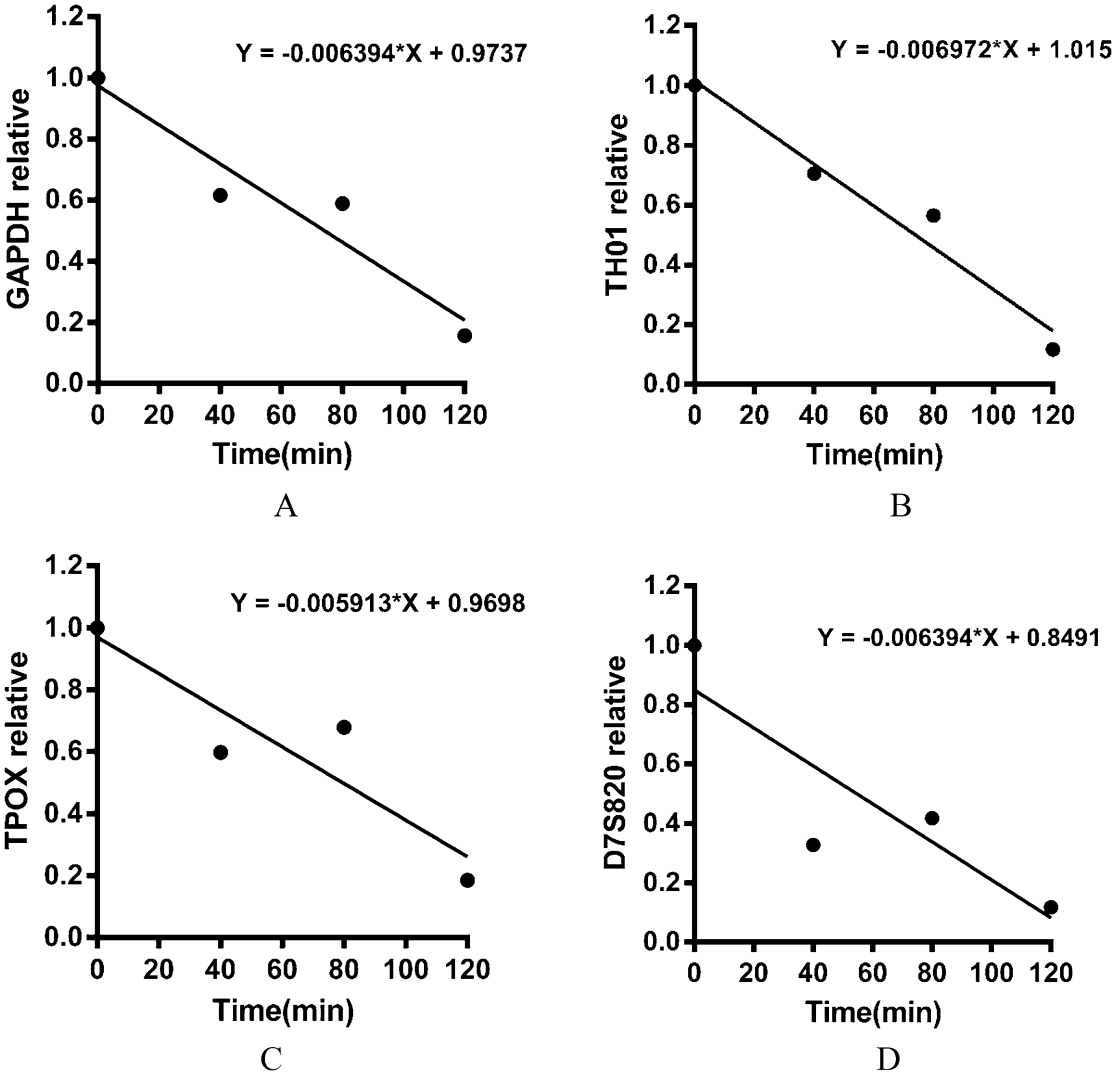
Analysis of the DNA degradation process and rate in the mouse gastrointestinal tract over time, on the basis of qPCR. (**A**), (**B**), (**C**) and (**D**) show the relatively quantitative linear relationship of the DNA of the four target genes with digestion time.

The relative quantification of the four targeted genes at different digestion times was calculated, as shown in Table 4 and Fig. 9. A significant decrease in the relative DNA concentration was observed at 40, 80 and 120 min post-gavage, as compared with pre-gavage concentrations (Table 4). At 120 min, an 85.62 ± 8.10% decrease in the concentration with respect to that pre-gavage was observed. According to the linear analysis, the digestion time and exogenous DNA degradation in the mouse stomach are shown in Fig. 10. The half-life of DNA degradation in the mouse stomach was 70.50 ± 5.46 min.

**Table 4:** Relative Ct values of DNA for four target genes in the mouse stomach at different digestion times.

Time (min)	GAPDH	TH01	TPOX	D7S820
0	1.0000	1.0000	1.0000	1.0000
40	0.6152	0.7043	0.5972	0.3271
80	0.5888	0.5647	0.6784	0.4175
120	0.1563	0.1169	0.1846	0.1174

**Figure 11:**
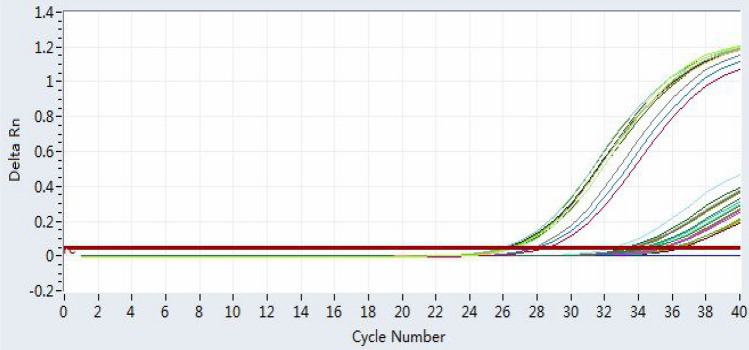
Amplification curves of the four target genes in the contents of the stomach, upper small intestine and lower small intestine in mice at 40 min after gavage. The red line in the figure is the baseline. The number of cycles required for PCR amplification to reach the baseline is called the threshold cycle (Ct) value. The smaller the Ct value, the higher the DNA concentration, and vice versa. In the figure, Ct values between 26 and 28 are shown for the four target genes amplified from mouse gastric content DNA; a Ct value around 35 is shown for the DNA concentration of the mouse small intestine content 40 min after gavage. When the Ct value reaches 35, almost no DNA template is present.

qPCR was used to detect the DNA amplification curve (Fig. 11), and the Ct values of the contents of different areas of the mouse GIT (stomach, upper small intestine and lower small intestine) at 40 min after gavage are shown in Table 5. From the amplification curve (Fig. 11), the Ct value of the mouse gastric contents at 40 min was approximately 26 (< 35). The Ct values of the four target genes in the upper part of the small intestine of the mice at 40 min were all less than 35; in the lower part of the small intestine of the mice at 40 min, only the GAPDH gene had a Ct value less than 35, and the Ct value of the other three target genes was greater than 35. Similarly, the target genes were not detected in the DNA of the small intestine contents in mice at the other times after gavage.

**Table 5:** Ct values of the four target genes from various region of digestive tract’s content of mice at 40 min after gavage.

Target gene	Threshold cycle (Ct)		
	Gastric content DNA	Small intestine chyme DNA (upper part)	Small intestine chyme DNA (lower part)
GAPDH	26.78	34.88	34.70
TH01	27.96	34.79	35.53
TPOX	26.16	33.65	35.29
D7S820	26.40	33.79	36.54

### STR results of DNA degradation in the mouse GIT

**Figure 12:**
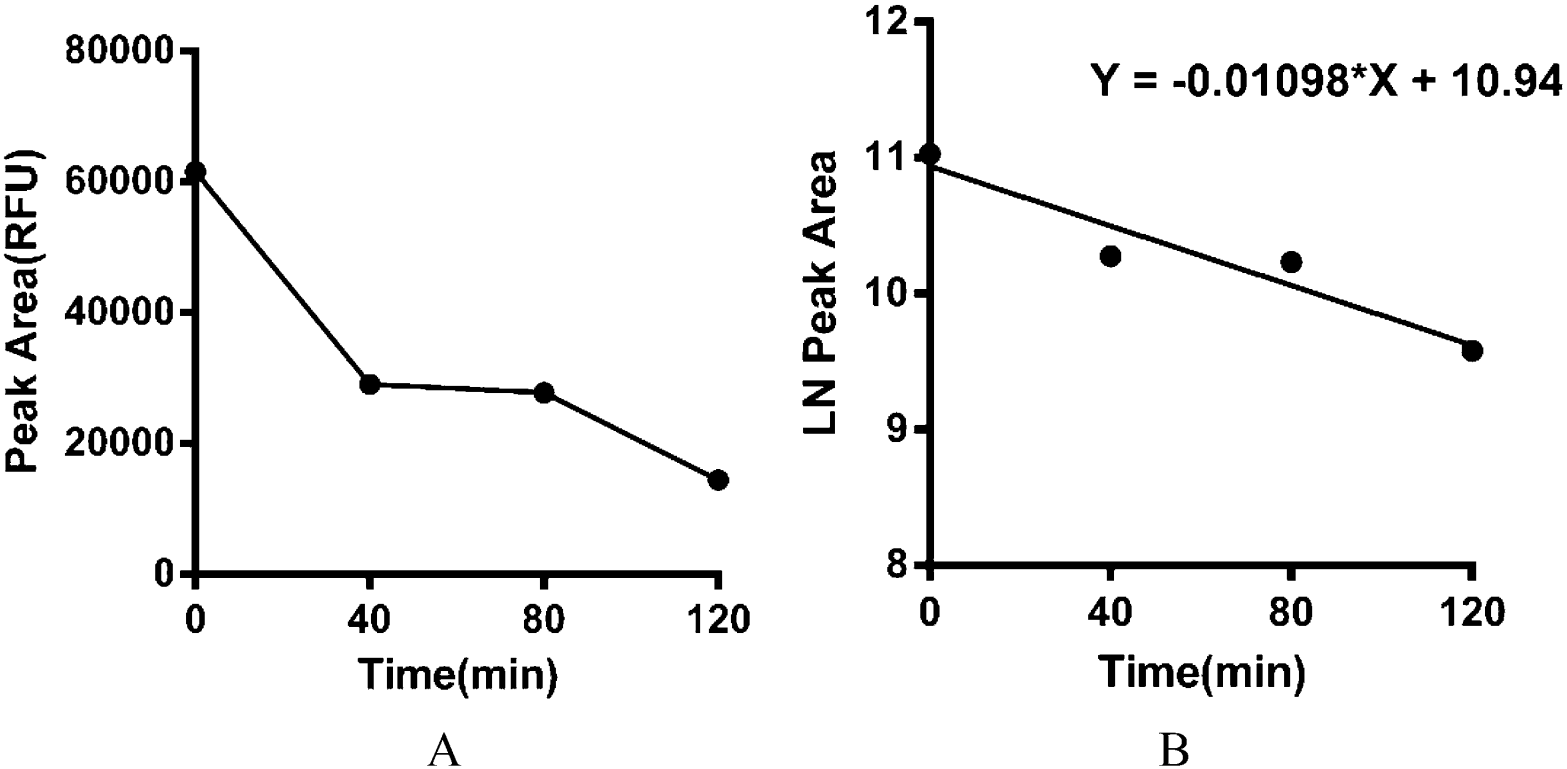
Analysis of the process and rate of DNA degradation in the mouse gastrointestinal tract over time, on the basis of STR assays. (**A**): average peak area of the stomach contents in mice at different digestion times. (**B**): linear regression analysis of the natural logarithm of the average peak area versus digestion time (half-life: 63.13 min).

The STR patterns of the DNA samples obtained from the mouse stomach at 0 min post-gavage are shown in Fig. 2. We used the average peak area as the concentrations of degraded DNA at different digestion times in the mouse stomach, as shown in Fig. 12A. The concentration of DNA decreased after digestion in the stomach in mice at 0 min. The concentration of DNA did not change significantly during 40–80 min and remained stable. After 120 min, the DNA concentration decreased significantly.

A linear regression was calculated with the natural logarithm of the average peak area versus digestion time, as shown in Fig. 12B. The degradation kinetic model was obtained. According to the formula: $$T\raise.5ex\hbox{$\scriptstyle 1$}\kern-.1em/ \kern-.15em\lower.25ex\hbox{$\scriptstyle 2$} = \ln 2/\left| k \right|$$, the half-life of DNA degradation in the mouse stomach was 63.13 min.

Through STR, the average peak area and the total number of alleles amplified were measured in various parts of the mouse digestive tract, and were 38, 4 and 1, in the stomach, upper small intestine and lower small intestine, respectively, at 40 min post gavage (Table 6). The results after 40 min were comparable, and over time, fewer alleles were amplified. The STR average peak area in different parts of the GIT decreased significantly with the advancement of bowel/chyme, as indicated by the decrease in the number of alleles detected in the stomach, upper and lower small intestine in mice.

**Table 6:** The average peak area and the number of alleles in the STR curve map of the DNA amplification of the contents of different parts of the mouse digestive tract at 40 min after gavage.

Samples	Average peak area (RFU)	Total alleles
Stomach content DNA	24010.42	38
DNA in upper small intestine contents	521.08	4
DNA in lower intestine contents	176.58	1

## Discussion

Food and medicine are vital to human survival. Hybrid food products are produced to feed humans and livestock. Recently nucleic acid treatment through parenteral or GIT administration has been reported^26^. The nucleic acid fate in the GIT tract has been unclear. A mouse model was developed to study the fate of DNA in the digestive tract in mice.

The advancement rate was found to be 20.27 ± 2.60% at 10 min post gavage. The advancement in the movement of bowel/chyme was observed in the GIT in mice. At 180 to 360 min post-gavage, the mouse stomach was completely emptied, and the contents of the stomach had fully entered the small intestine. This finding is consistent with those reported by Chul-Hyun Lim^27^, who used a ^13^C octanoic acid breath test to measure gastric emptying times in mice. After 510 to 570 min of digestion, all contents had entered the cecum. This finding aids in the understanding of bowel movement advancement.

Currently, the most commonly used DNA-based detection techniques are PCR and qPCR. qPCR, the most commonly used DNA quantification method, has the advantages of low pollution and high specificity^28^. As shown in Figs. 6 and 7, clear bands in the mouse stomach were observed pre-gavage (0 min) and post-gavage (i.e., during 40–120 min). These results confirmed the presence of human leukocyte DNA in the stomach in mice after 120 min of digestion (> 200 bp). There were no observed DNA bands for the four target genes in the contents of the small intestine in mice at 40, 80 and 120 min after gavage; therefore, we believe that the DNA had degraded into fragments of < 77 bp in the small intestine in the mice. Nawaz^15^ found that food DNA can survive in the digestion process, and DNA fragments up to several hundred bp can be detected in the GIT. In agreement with prior study findings^9–13^, DNA fragments were clearly detected in the GIT of animals; however, whether they can be detected in the blood and other tissues requires further experiments.

Anatomically, the mouse stomach consists of two regions—the non-glandular/fore-stomach and glandular stomach—which are separated by a limiting ridge^29^. Gastric juice consists of pepsin and gastric acid. Pepsin's main function is to digest protein, but in recent years, Liu^17^ found that the pepsin in gastric juice can digest not only protein but also nucleic acid. The digestion of nucleic acid starts in the stomach, and various animal pepsins have different abilities to digest nucleic acid^18^. Most dietary DNA is in the form of histones, which form nucleosomes. The complex components of the diet may affect the digestion of DNA by pepsin. Zhang^19^ demonstrated that common food components, including proteins, carbohydrates, metal cations and polycationic compounds, are closely associated with the digestion of DNA through in vitro simulation studies.

Ct values in the gastric contents of mice at 0, 40, 80 and 120 min after gavage were measured through qPCR, as shown in Fig. 8. The DNA concentration decreased consistently from 0 to 120 min post gavage, and at 120 min, an 85.62 ± 8.10% decrease with respect to 0 min was observed (Table 4). The half-life of DNA degradation in the mouse stomach was 70.50 ± 5.46 min (Fig. 10). This finding indicated that the DNA concentration in the mouse stomach decreased significantly. Wiedemann^30^ analyzed the rubisco and cry1Ab genes through real-time PCR and reported degradation of 20% of the initial value at 2 h. The degradation was 0.5% of the initial value after incubation for 48 h in the rumen.

DNA degradation may be associated with mechanical aspects, gastric juice and microorganisms in the mouse stomach. The DNA was not completely degraded in the stomach in mice, and > 200 bp DNA fragments remained. Protein and carbohydrate, the main components of food, do not affect DNA digestion at the concentrations recommended by the WHO (40:1 and 80:1). When the ratio of protein to DNA is > 80:1, DNA digestion is inhibited^18^. Divalent cations (Ca2^+^ and Mg^2+^) can result in greater DNA digestion than monovalent cations (Na^+^ and K^+^)^18^. The gavage included nutritive semi-solid paste and human white blood cells. The sodium carboxymethyl cellulose, starch and milk powder in the nutritive semi-solid paste resembled normal dietary components, thus potentially inhibiting DNA digestion. In addition, the structure of human leukocytes includes a cell membrane and nucleus, which may protect against DNA digestion. According to Zhang, pepsin has a digestive effect toward nucleic acid, on the basis of in vitro simulation: pepsin can digest specific sequences nucleic acids, such as 5´-AAG↓AA-3´ and CGA↓T^17^. The target genes TH01, TPOX and D7S820 have repetitive sequences rich in TCAT, GAAT and GATA, respectively. Mouse pepsin may have a restriction enzymatic effect on these sequences, thus resulting in DNA degradation.

In qPCR method, when the Ct value is > 35, the target gene is considered absent. In Table 5, from the amplification curve, the Ct value of the mouse gastric contents at 40 min was approximately 26 (< 35). The Ct values of the four target genes in the upper part of the small intestine of the mice at 40 min were all less than 35, thus indicating that the four target genes were present in very low amounts; in the lower part of the mouse small intestine at 40 min, only the GAPDH gene had a Ct value less than 35. These results indicated the presence of a small amount of GAPDH, whereas the Ct values of the other three target genes were > 35, indicating the absence of the target genes. Similarly, the target genes were not detected in the DNA of the small intestine contents in mice at other times after gavage (Fig. 11). These results were consistent with the PCR results, indicating that DNA was further degraded into small fragments < 77 bp in the small intestine by digestive enzymes and intestinal microorganisms. The DNA in human white blood cells was more easily degraded digested by the gastric juices in mice when it entered the intestines.

The STR technique was used to amplify small fragments of DNA. The average peak areas of 21 STR loci amplified by PCR have been found to provide a good representation of DNA degradation^22^. The capillary zone electrophoresis-laser induced fluorescence method can be used to determine the DNA concentrations in serum and plasma, and is as accurate and sensitive as the widely used real-time PCR method^31,32^.

The results obtained from plotting the average peak area and gavage time were consistent with the results of qPCR (Fig. 12A). According to the natural logarithm of the average peak area versus the digestion time, the half-life of DNA degradation in the mouse stomach was 63.13 min. This result was consistent with the half-life of DNA degradation determined by qPCR. After food enters the stomach, through mechanical digestion and chemical digestion^33^, part of the food bolus enters the intestines in the form of chyme. Our results showed that the rate of cellular DNA degradation in the mouse stomach was slow. STR map analysis of DNA in the small intestine in mice revealed that as the chyme advances in the small intestine, the number of human DNA alleles decreased (Table 6). qPCR and STR both clearly showed that human genomic DNA was markedly more degraded in the mouse intestine than the mouse stomach, whereas human target genes were degraded gradually in the mouse intestine with chyme advancement. Gene degradation times were also predicted through the STR method (Fig. 12B).

Liu^19^ et al. reported that nucleic acids are digested in the stomach in blackhead fish and banded grouper, whereas the digestion of nucleic acids by bovine gastric enzymes was not observed. Different animals have varying ability to digest nucleic acids with pepsin. According to the experimental results of the current study, the mouse pepsin can be assumed to have a digestive effect toward human genomic DNA, thus, providing a potential reference for future experiments.

In recent years, no detailed research has performed a quantitative analysis of the degradation of DNA in the digestive tract in mice. Our findings should contribute to future food and drug research, forensics and risk assessment of genetically modified foods.

## Conclusions and recommendations

The degradation of exogenous DNA was higher in the mouse stomach during first 2 h, and almost complete degradation was observed within 40 min after entering the small intestine in mice.

We observed the digestive kinetics in mice in vivo, and this information may be valuable in future experimental studies. However, because this experiment was based on observation of the mouse gastrointestinal tract after sacrifice, the time intervals were all integers; to refine the findings, further research and improvement are needed.

## Supplementary Information

Supplementary Information.
