# Supplementary material for: Measuring the process and rate of exogenous DNA degradation during digestion in mice
Source: Sci Rep. 2022 Apr 19;12:6463. doi: 10.1038/s41598-022-10340-7 (PMC9018913; doi:10.1038/s41598-022-10340-7)
Supplement: Supplementary file 1 — Supplementary Information. [file 41598_2022_10340_MOESM1_ESM.docx]

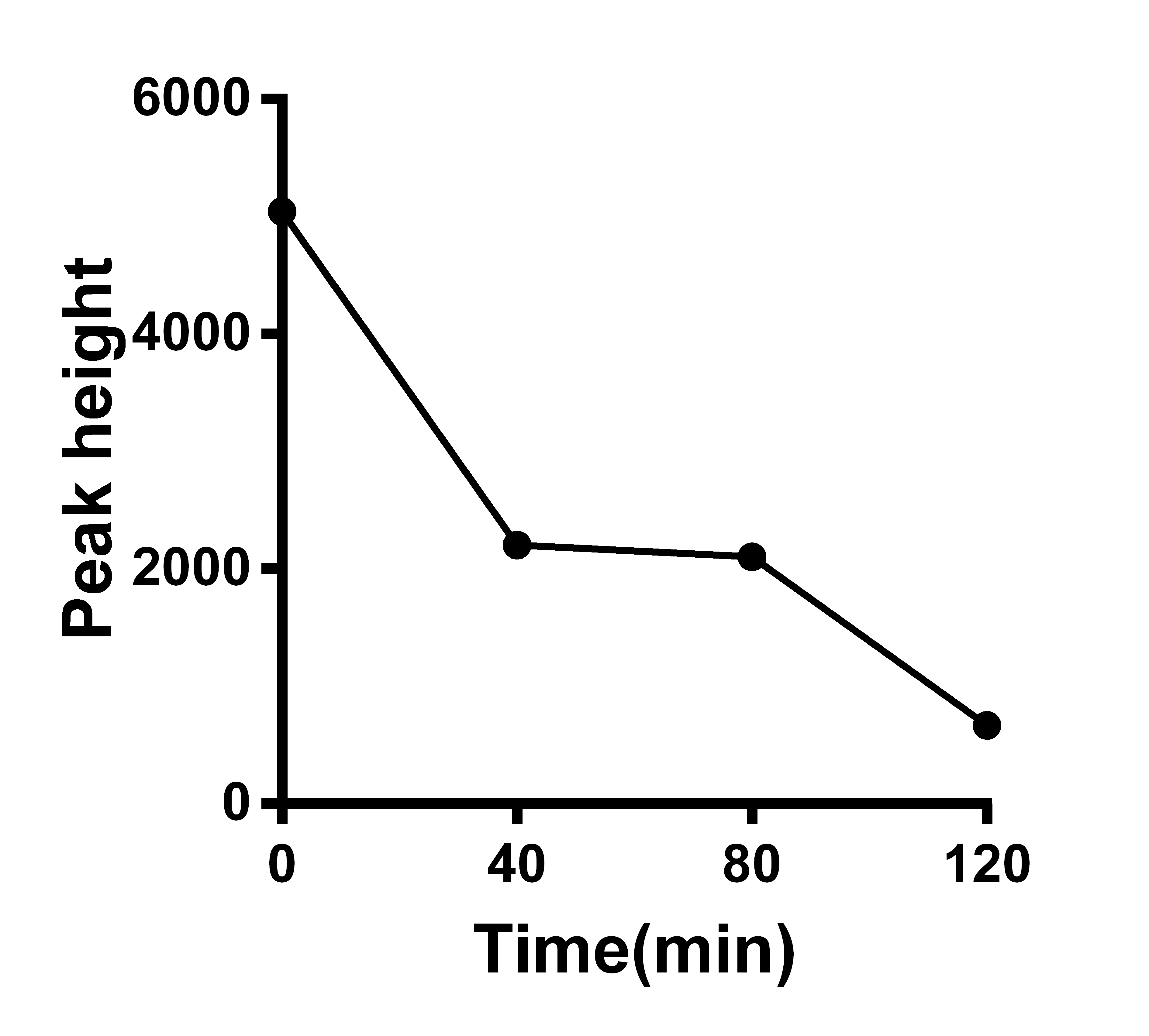


S1：average peak hight of the stomach contents in mice at different digestion times.


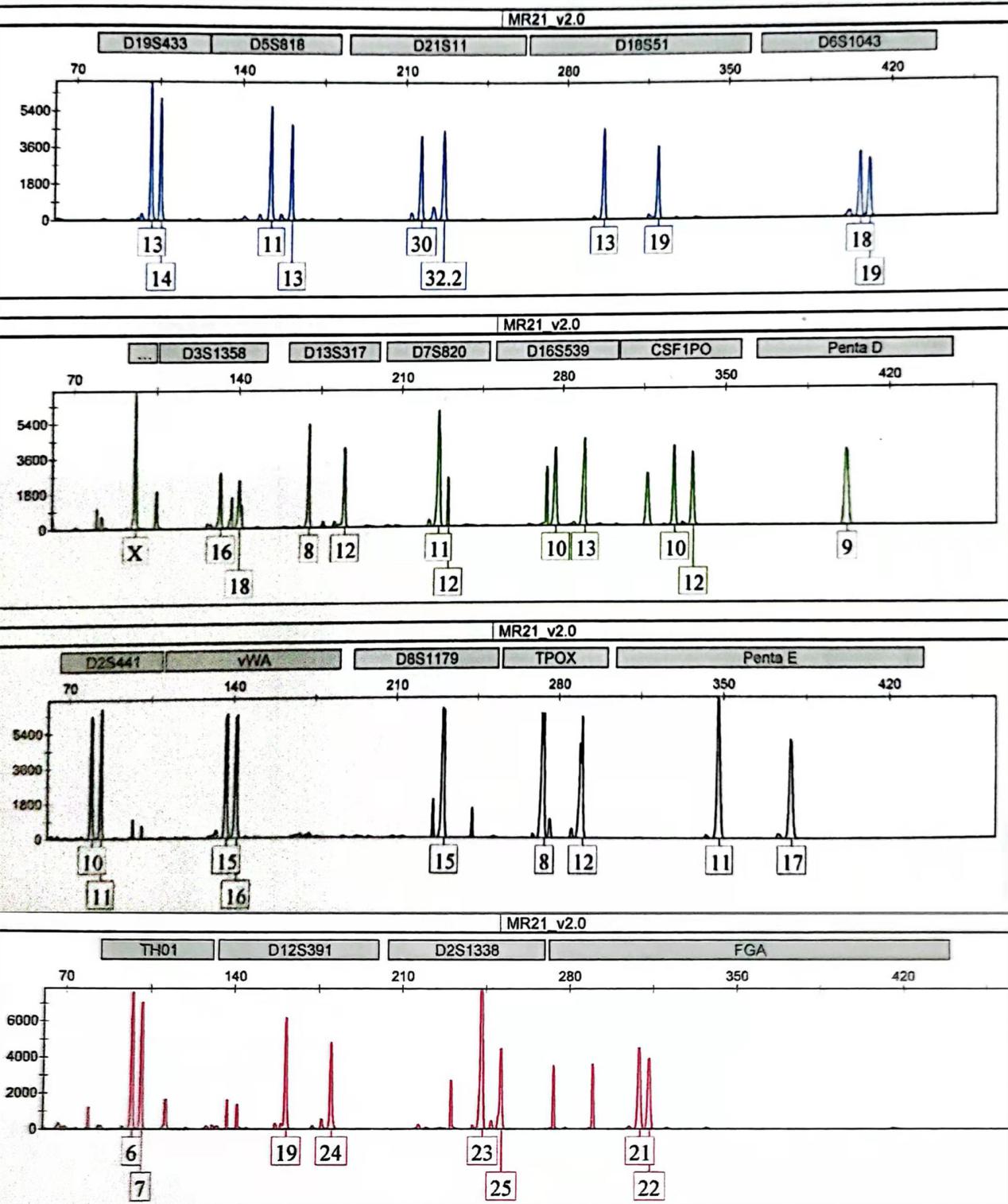
S2: STR profile of DNA in gastric contents of mice after gavage for 0 minutes


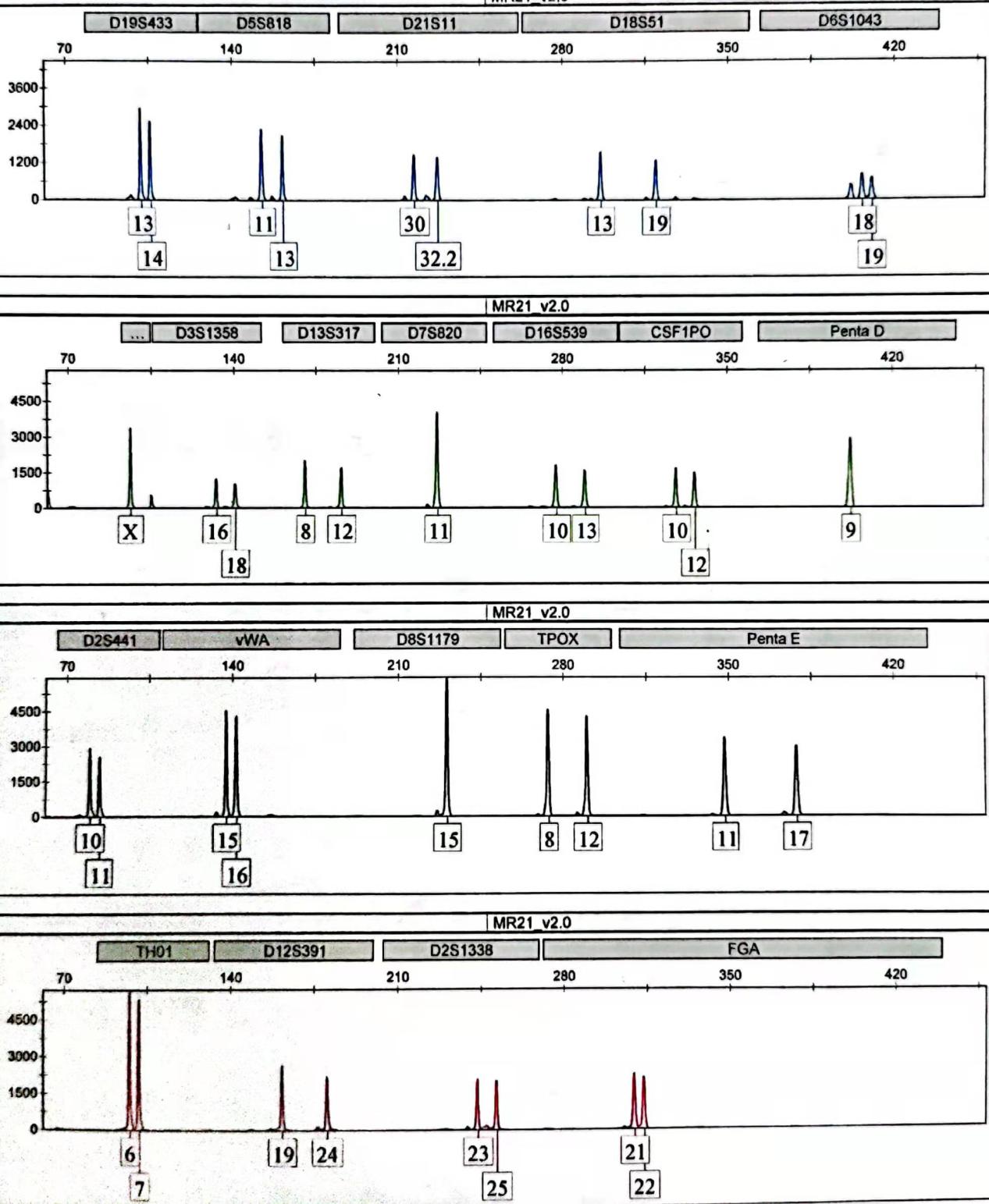


S3: STR profile of DNA in gastric contents of mice after gavage for 40 minutes


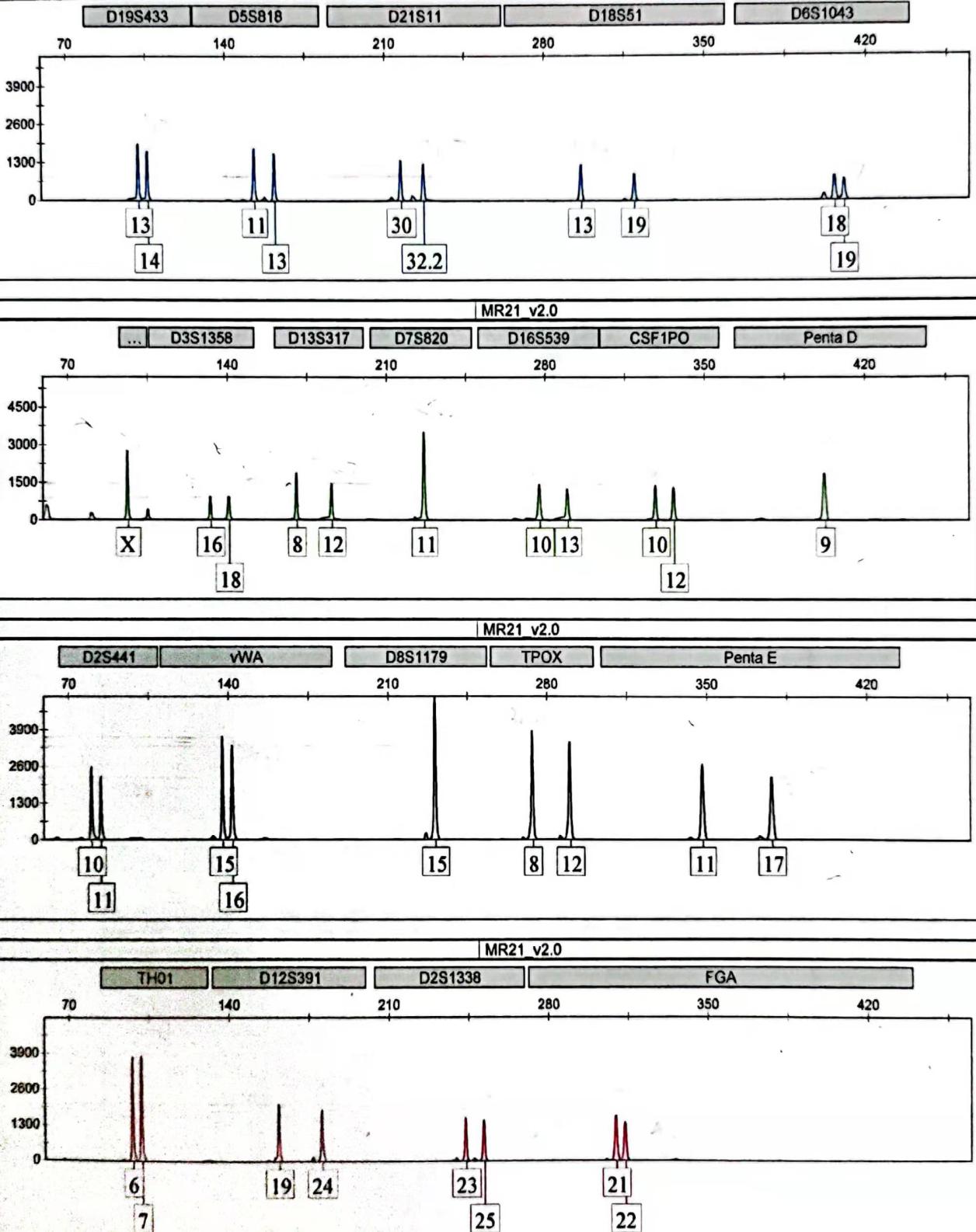


S4: STR profile of DNA in gastric contents of mice after gavage for 80 minutes


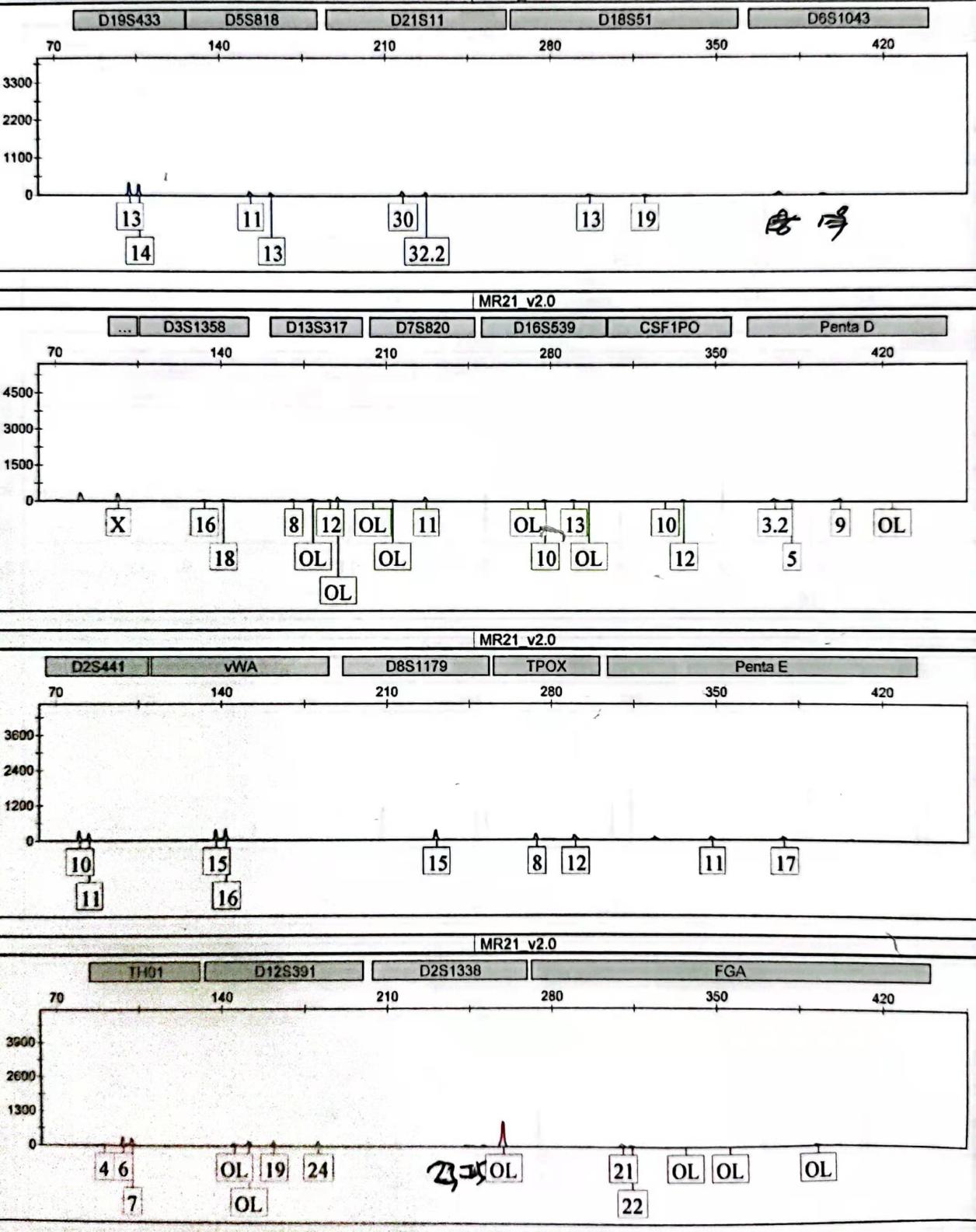


S5: STR profile of DNA in gastric contents of mice after gavage for 120 minutes


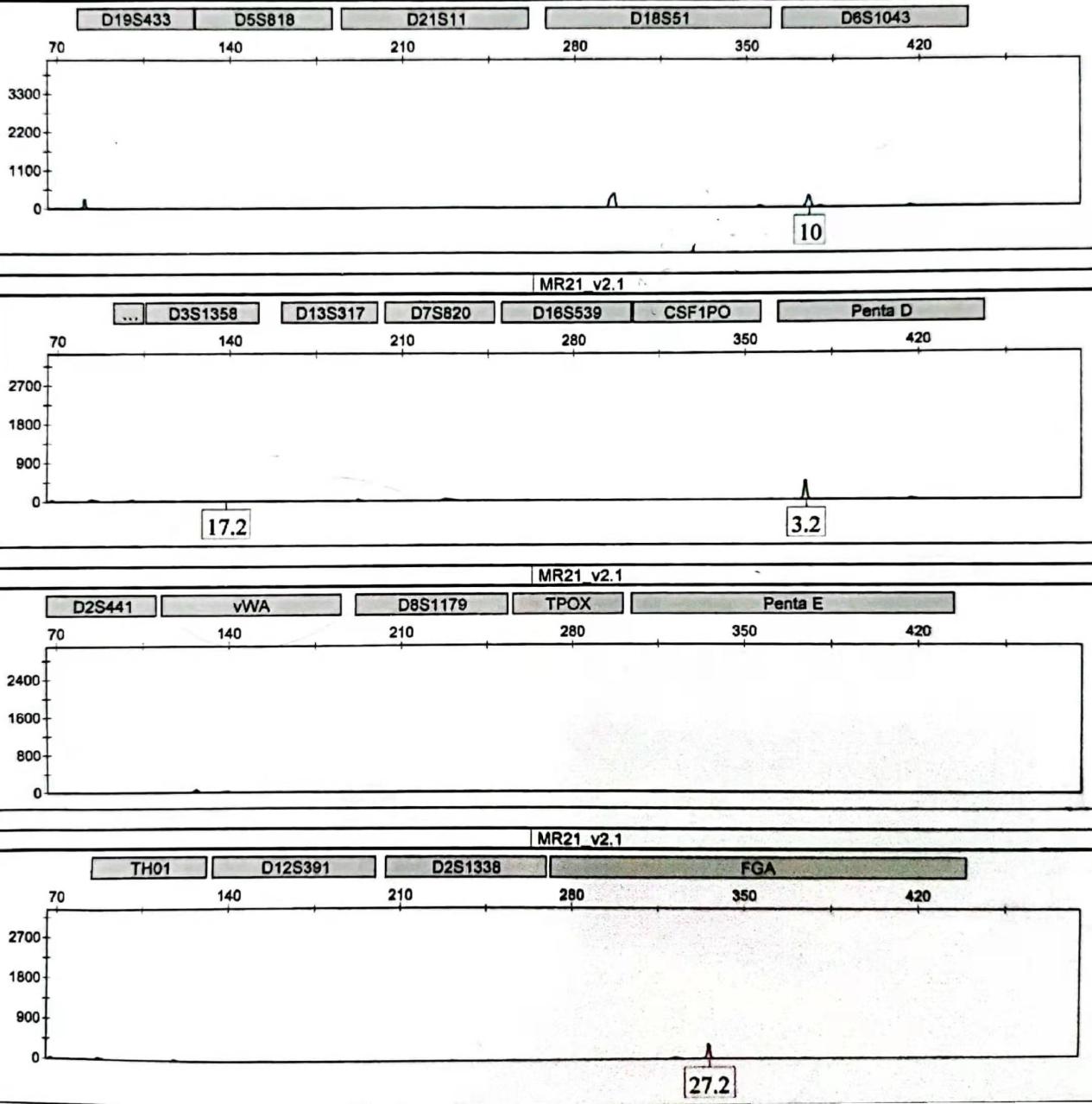


S6: STR profiles of DNA in the upper small intestine of mice after gavage for 40 minutes
